# Rb_2_Lu[Si_4_O_10_]F, a tubular chain silicate

**DOI:** 10.1107/S1600536814003043

**Published:** 2014-02-19

**Authors:** Volker Kahlenberg, Tanja Manninger

**Affiliations:** a University of Innsbruck, Institute of Mineralogy & Petrography, Innrain 52, A-6020 Innsbruck, Austria

## Abstract

Single crystals of Rb_2_Lu[Si_4_O_10_]F (dirubidium lutetium tetra­silicate fluoride) were obtained in flux-synthesis experiments in the system SiO_2_–Lu_2_O_3_–RbF. The compound belongs to the group of tubular chain silicates, *i.e.* it is based on multiple chains of condensed [SiO_4_] tetra­hedra forming closed columns. The periodicity of the unbranched multiple chains is four and corresponds to the length of the *b* axis. Adjacent columns are connected by Lu^3+^ cations, which are coordinated by four oxide and two fluoride anions in the form of slightly distorted octa­hedra. By sharing common fluoride corners, the single octa­hedra are linked into chains running parallel to the silicate tubes. Electroneutrality is achieved by the incorporation of additional Rb^+^ cations. All four symmetrically independent rubidium ions, four out of twelve oxide as well as the two fluoride anions are located on mirror planes. The remaining atoms reside on general positions.

## Related literature   

Oxosilicates that contain monovalent alkali cations, trivalent rare earth elements and additional fluorine anions have potential application in the field of luminescense (Jacobsen & Meyer, 1994 ▶; Tang *et al.*, 2008 ▶; Schäfer & Schleid, 2007 ▶, 2011 ▶; Kahlenberg & Manninger, 2014 ▶). For structures isotypic to that of the title compound, see: Chigarov *et al.* (1983 ▶); Hung *et al.* (2003 ▶). For general aspects of the crystal chemistry of silicates, see: Liebau (1985 ▶). For the definition of distortion parameters, see: Robinson *et al.* (1971 ▶). For bond-valence analysis, see: Brown (2002 ▶). For the definition and calculation of similarity descriptors, see: Tasci *et al.* (2012 ▶); Bergerhoff *et al.* (1999 ▶). For the Inorganic Structure Database, see: ICSD (2014 ▶).

## Experimental   

### 

#### Crystal data  

Rb_2_Lu[Si_4_O_10_]F
*M* *_r_* = 637.27
Monoclinic, 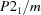

*a* = 11.6695 (3) Å
*b* = 8.52379 (18) Å
*c* = 11.8165 (3) Å
β = 111.753 (3)°
*V* = 1091.67 (5) Å^3^
*Z* = 4
Mo *K*α radiation
μ = 18.4 mm^−1^
*T* = 298 K
0.32 × 0.08 × 0.08 mm

#### Data collection  

Oxford Diffraction Xcalibur (Ruby, Gemini ultra) diffractometer
Absorption correction: analytical [*CrysAlis PRO* (Oxford Diffraction, 2006 ▶), based on expressions derived by Clark & Reid (1995 ▶)] *T*_min_ = 0.106, *T*_max_ = 0.562
15185 measured reflections
2388 independent reflections
2276 reflections with *I* > 2σ(*I*)
*R*_int_ = 0.028

#### Refinement  

*R*[*F* ^2^ > 2σ(*F* ^2^)] = 0.017
*wR*(*F* ^2^) = 0.037
*S* = 1.2
2388 reflections
178 parameters
Δρ_max_ = 0.67 e Å^−3^
Δρ_min_ = −0.69 e Å^−3^

### 

Data collection: *CrysAlis PRO* (Oxford Diffraction, 2006 ▶); cell refinement: *CrysAlis PRO*; data reduction: *CrysAlis PRO*; program(s) used to solve structure: *SIR2002* (Burla *et al.*, 2003 ▶); program(s) used to refine structure: *SHELXL97* (Sheldrick, 2008 ▶); molecular graphics: *ATOMS for Windows* (Dowty, 2011 ▶); software used to prepare material for publication: *publCIF* (Westrip, 2010 ▶) and *WinGX* (Farrugia, 2012 ▶).

## Supplementary Material

Crystal structure: contains datablock(s) global, I, New_Global_Publ_Block. DOI: 10.1107/S1600536814003043/wm5002sup1.cif

Structure factors: contains datablock(s) I. DOI: 10.1107/S1600536814003043/wm5002Isup2.hkl

CCDC reference: 986192

Additional supporting information:  crystallographic information; 3D view; checkCIF report

## Figures and Tables

**Fig. 1.**
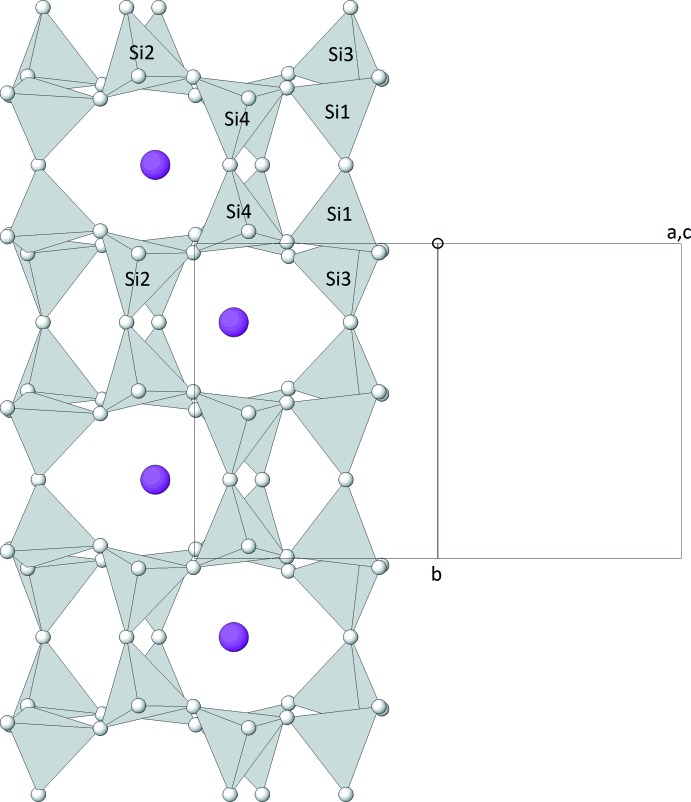
A single tubular chain consisting of [SiO4]-tetrahedra in a projection parallel to [101]. Large blue spheres represent Rb2 cations.

**Fig. 2.**
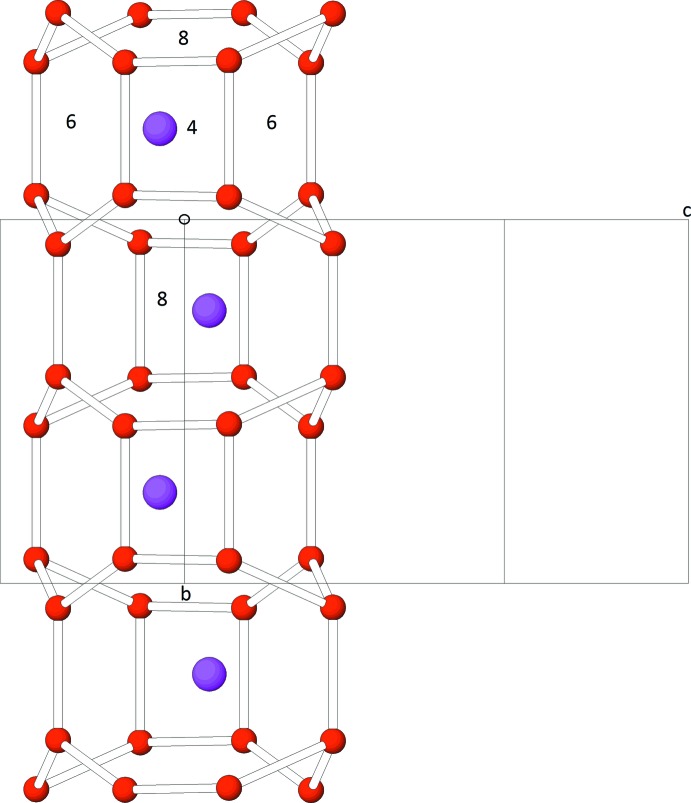
Connectivity of the silicon atoms within a single tubular chain (view perpedicular to (011)). Red spheres represent the Q3- connected Si atoms. Large blue spheres represent Rb2 cations. The sizes of the different ring types are indicated.

**Fig. 3.**
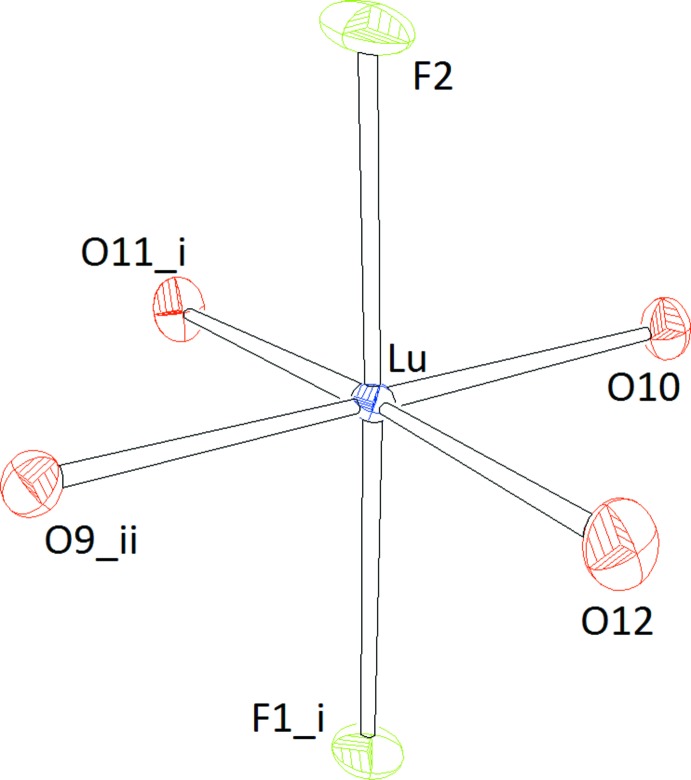
Representation of the coordination polyhedron around Lu. Ellipsoids are drawn at the 60% level. [Symmetry codes: (i) x, y - 1, z; (ii) -x + 1, -y, -z + 1.]

**Fig. 4.**
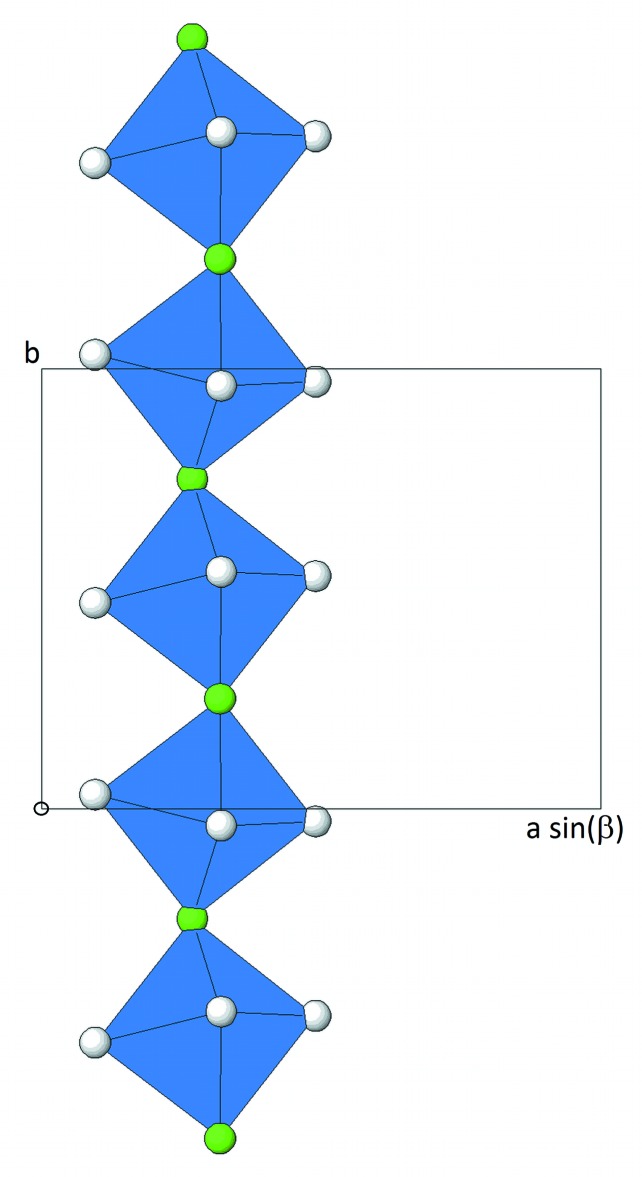
Projection of a single chain of corner-sharing [LuO4F2]-octahedra parallel to [001]. Small grey and green spheres represent oxygen and fluoride anions, respectively.

**Fig. 5.**
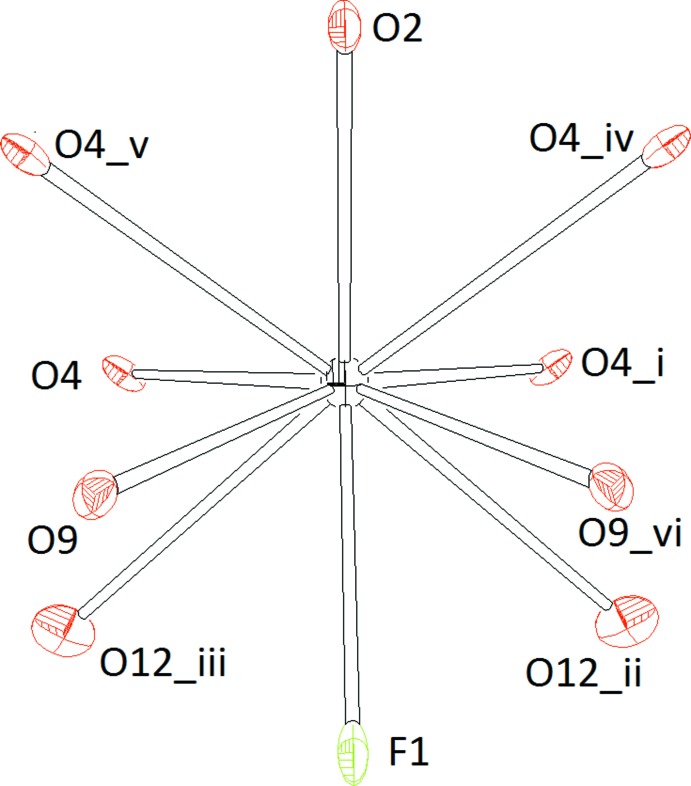
Representation of the coordination polyhedron around Rb1. Ellipsoids are drawn at the 60% probability level. [Symmetry codes: (i) x, 3/2 - y, z; (ii) x, 1/2 - y, z; (iii) x, 1 + y, z; (iv) 1 - x, -1/2 + y, 1 - z; (v) 1 - x, 2 - y, 1 - z; (vi) 1 - x, 1/2 + y, 1 - z.]

**Fig. 6.**
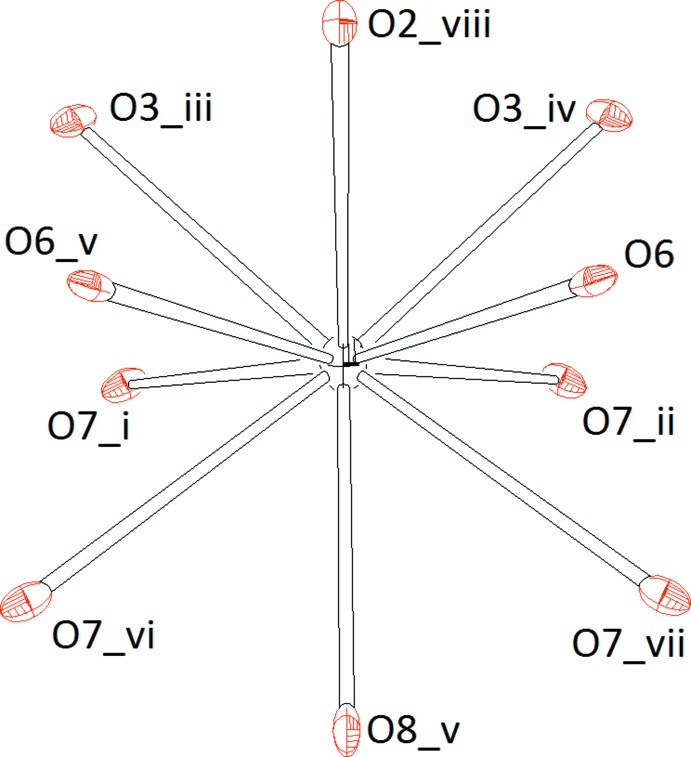
Representation of the coordination polyhedron around Rb2. Ellipsoids are drawn at the 60% probability level. [Symmetry codes: (i) x, 1/2 - y, -1 + z; (ii) x, y, -1 + z; (iii) x, 3/2 - y, z; (iv) x, -1 + y, z; (v) -x, -1/2 + y, 1 - z; (vi) -x, 1/2 + y, 1 - z; (vii) -x, -y, 1 - z; (viii) 1 - x, -1/2 + y, 1 - z.]

**Fig. 7.**
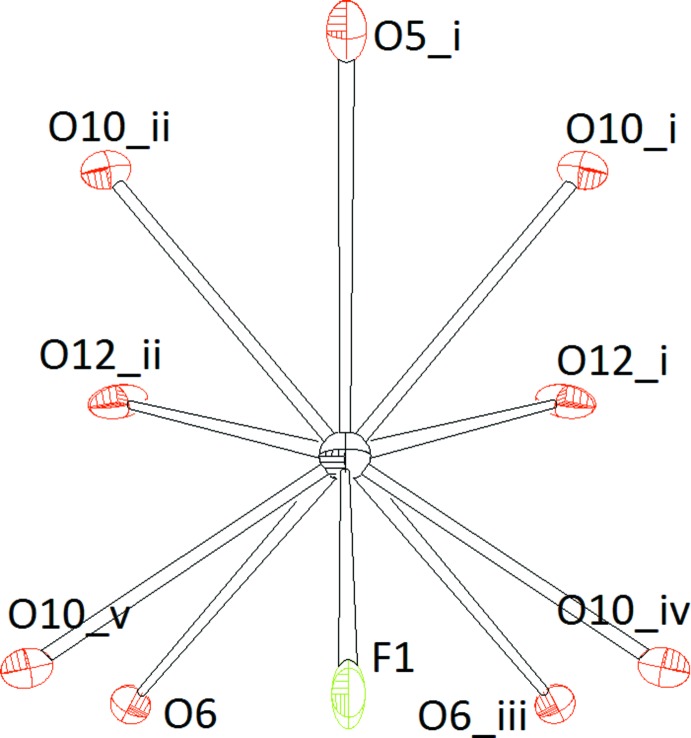
Representation of the coordination polyhedron around Rb3. Ellipsoids are drawn at the 60% probability level. [Symmetry codes: (i) -x, 1/2 + y, 1 - z; (ii) -x, 1 - y, 1 - z; (iii) x, 3/2 - y, z; (iv) x, 1/2 - y, z; (v) x, 1 + y, z.]

**Fig. 8.**
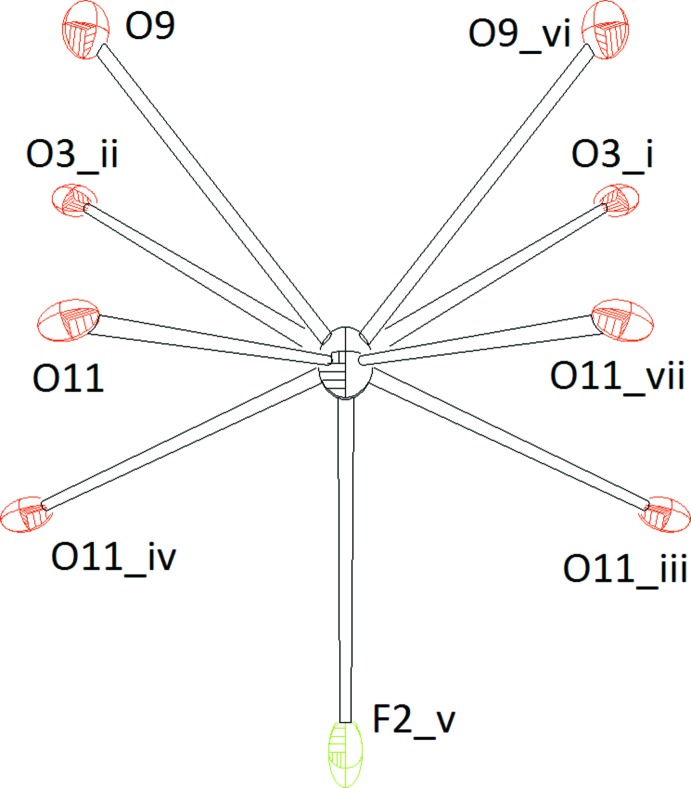
Representation of the coordination polyhedron around Rb4. Ellipsoids are drawn at the 60% probability level. [Symmetry codes: (i) x, 3/2 - y, z; (ii) x, -1 + y, z; (iii) x, 3/2 - y, -1 + z; (iv) x, -1 + y, -1 + z; (v) x, y, -1 + z; (vi) x, 1/2 - y, z; (vii) 1 - x, -1/2 + y, 1 - z.]

**Fig. 9.**
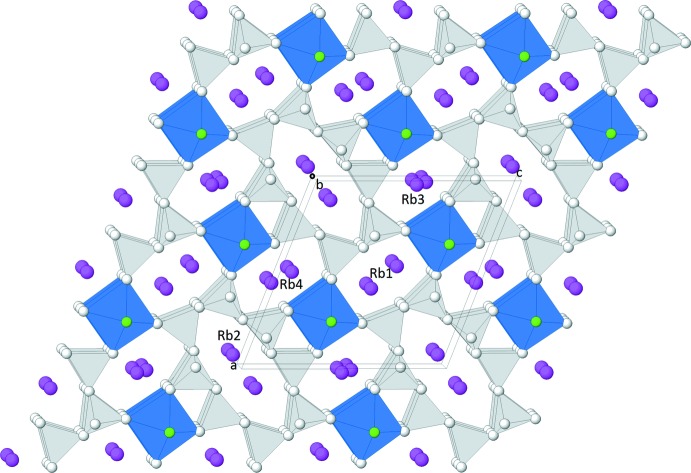
Side view of the crystal structure of Rb2Lu[Si4O10]F. [SiO4]- and [LuO4F2]-polyhedra are shown in light-grey and blue. Small grey spheres represent oxygen atoms. Fluoride and rubidium ions are given as green and pink spheres, respectively.

## supplementary crystallographic information

### 1. Comment

Up to now several alkali-*REE*-fluoride silicates (*REE* is a rare earth metal) including compounds such as KEu_2_[Si_4_O_10_]F (Jacobsen & Meyer, 1994), K_9_(*REE*)_3_[Si_12_O_32_]F_2_ (Tang *et al.*, 2008; Kahlenberg & Manninger, 2014), Cs_2_Y[Si_4_O_10_]F_2_ (Schäfer & Schleid, 2007) or Rb_3_Sc_2_[Si_4_O_10_]F_5_ (Schäfer & Schleid, 2011) have been reported. In the course of an ongoing project on the synthesis of new representatives of this class, single-crystals of the previously unknown compound Rb_2_Lu[Si_4_O_10_]F have been structurally characterized. Following Liebau's classification of oxosilicates (Liebau, 1985), the crystal structure of this phase belongs to the group of tubular chain silicates and is based on unbranched multiple silicate chains running along [010] (Fig. 1). The periodicity of the chains is four. Alternatively, the tubular chains can also be thought of as being built from the condensation of an infinite number of fundamental rings with mean planes perpendicular to the chain direction (Liebau, 1985). In Rb_2_Lu[Si_4_O_10_]F these rings are eight-membered (Fig. 2) and exhibit a twisted chair conformation. Within the tubes, cages can be identified that are formed by additional four-, six- and eight-membered rings, the mean planes of which are running parallel to the chains. These six- and eight-membered rings are in boat configurations.

According to the Si:O ratio of 1:2.5 the structure is exclusively based on tertiary (*Q*^3^) [SiO_4_] tetrahedra. This structural feature is also reflected in the spread of the Si—O bond lengths. Each of the four crystallographically independent tetrahedra has one short (1.569 (3)–1.581 (3) Å) Si—O bond involving the non-bridging O atoms. The distances between Si and the bridging O atoms are considerably longer. The O–Si–O angles show a significant scatter throughout all present [SiO_4_] tetrahedra. Nevertheless, the values are in the expected limits for silicates (Liebau, 1985). Numerically, the degree of distortion can be expressed by the quadratic elongation λ and the angle variance σ^2^ (Robinson *et al.*, 1971). For the four tetrahedra these two parameters vary between 1.001 and 1.005 (for λ) and 5.29 and 23.24 (for σ^2^) indicating that the deviation from regularity is not very pronounced. The Lu^3+^ cations are octahedrally coordinated by four oxygen and two additional fluoride anions (Fig. 3). The latter two are in *trans*-configuration. By sharing common fluorine corners, the octahedra in turn form chains running parallel to the directions of the silicate tubes (Fig. 4). However, these chains are not straight. The polyhedra are tilted with tilt angles (Lu—F1—Lu) and (Lu—F2—Lu) of 160.5 (3) and 156.3 (3)°, respectively. Charge compensation is achieved by the incorporation of additional Rb^+^ ions. The coordination numbers of these cations are as follows: Rb1, Rb3: 10-coordinate, including one F atom each; Rb2: 10-coordinate; Rb4: 9-coordinate, including one F atom (Fig. 5–8). The Rb2 cations, which are exclusively coordinated by O atoms, are located within the abovementioned cages of the tubes. The remaining rubidium cations reside in tunnel-like cavities formed by [SiO_4_]-tetrahedra and [LuO_4_F_2_]-octahedra. A side view of the crystal structure is given in Fig. 9.

Bond valence sum calculations using the parameter sets for the Rb–O, Rb–F, Lu–O, Lu–F and Si–O bonds given by Brown (2002) resulted in the following values (in v.u.) for the cation-anion interactions up to 3.4 Å: Rb(1): 1.152, Rb(2): 1.324, Rb(3): 1.008, Rb(4): 0.936, Lu: 3.140, Si(1): 4.062, Si(2): 4.044, Si(3): 4.082 and Si(4): 4.028.

The present compound is isostructural with K_2_Lu[Si_4_O_10_](OH) (Chigarov *et al.*, 1983) and K_2_In[Si_4_O_10_](OH) (Hung *et al.*, 2003). For the calculation of several quantitative descriptors for the characterization of the degree of similarity between the crystal structures of Rb_2_Lu[Si_4_O_10_]F and K_2_Lu[Si_4_O_10_](OH) containing the same *REE*, the program *COMPSTRU* (Tasci *et al.*, 2012) was employed. For the two structures, the degree of lattice distortion (S), *i.e.* the spontaneous strain obtained from the eigenvalues of the finite Lagrangian strain tensor calculated in a Cartesian reference system, has a value of (S) = 0.0053. For further investigations on an atomic level, the proton positions of the hydroxyl groups in K_2_Lu[Si_4_O_10_](OH) have been neglected. After a transformation to the standard setting according to a' = b, b' = c and c' = a and the application of an origin shift of p = (0, 1/2, 1/2) the structure of K_2_Lu[Si_4_O_10_](OH) was mapped on the most similar configuration of Rb_2_Lu[Si_4_O_10_]F. The calculations revealed the following atomic displacements (in Å) between the corresponding atoms in Rb_2_Lu[Si_4_O_10_]F (first entry) and K_2_Lu[Si_4_O_10_](OH) (second entry): Rb1—K1: 0.091; Rb2—K3: 0.063; Rb3—K2: 0.275; Rb4—K4: 0.064; Lu—Lu: 0.098; Si1—Si1: 0.055; Si2—Si2: 0.114; Si3—Si4: 0.089; Si4—Si3: 0.095; O1—O4: 0.083; O2—O13: 0.134; O3—O9: 0.067; O4—O5: 0.207; O5—O3: 0.168; O6—O11: 0.149; O7—O10: 0.101; O8—O2: 0.116; O9—O7: 0.082; O10—O14: 0.173; O11—O6: 0.247; O12—O8: 0.134; F1—O1: 0.246; F2—O12: 0.125, *i.e.* the maximum displacement is 0.275 Å. The measure of similarity (Δ) as defined by Bergerhoff *et al.* (1999) has a value of 0.022.

### 2. Experimental

Single-crystals of Rb_2_Lu[Si_4_O_10_]F were obtained in the course of a series of flux syntheses experiments aiming on the preparation of new Rb-*REE*-fluoride silicates. 0.1 g of the nutrient consisting of a mixture of Lu_2_O_3_:SiO_2_ in the molar ratio 1:4 was homogenized in an agate mortar with 0.1 g RbF, transferred into a platinum tube and welded shut. The container was heated in a laboratory chamber furnace from 373 K to 1373 K with a ramp of 50 K/h and isothermed for 2 h at the target temperature. Subsequently, the sample was cooled down to 1073 K with a rate of 5 K and, finally, the temperature was reduced to 373 K with a rate of 100 K/h. After the removal of the platinum tube the solidified melt cake was immediately crashed in an agate mortar and transferred to a glass slide under a polarizing binocular. A first optical inspection revealed the presence of two phases: a polycrystalline matrix of RbF in which transparent birefringent single-crystals up to 400µm in size were embedded. One of the optically biaxial crystals showing sharp extinction when observed between crossed polarizers was selected for further structural studies and was mounted on the tip of a glass fiber using fingernail hardener as glue.

### 3. Refinement

Similar sets of lattice parameters could be found in the recent WEB-based version of the Inorganic Crystal Structure Database (ICSD, 2014) for the chemically closely related compounds K_2_Lu[Si_4_O_10_]F (Chigarov *et al.*, 1983) and K_2_In[Si_4_O_10_](OH) (Hung *et al.*, 2003) pointing to isostructural relationships, which were confirmed by subsequent structure analysis. A data set corresponding to a hemisphere of reciprocal space was collected.

### Crystal data

**Table:**

Rb_2_Lu[Si_4_O_10_]F	*F*(000) = 1160
*M**_r_* = 637.27	*D*_x_ = 3.877 Mg m^−^^3^
Monoclinic, *P*2_1_/*m*	Mo *K*α radiation, λ = 0.71073 Å
Hall symbol: -P 2yb	Cell parameters from 8764 reflections
*a* = 11.6695 (3) Å	θ = 3.0–29.4°
*b* = 8.52379 (18) Å	µ = 18.4 mm^−^^1^
*c* = 11.8165 (3) Å	*T* = 298 K
β = 111.753 (3)°	Prism, colourless
*V* = 1091.67 (5) Å^3^	0.32 × 0.08 × 0.08 mm
*Z* = 4	

### Data collection

**Table:**

Oxford Diffraction Xcalibur (Ruby, Gemini ultra) diffractometer	2388 independent reflections
Graphite monochromator	2276 reflections with *I* > 2σ(*I*)
Detector resolution: 10.3575 pixels mm^-1^	*R*_int_ = 0.028
ω scans	θ_max_ = 26.4°, θ_min_ = 3.0°
Absorption correction: analytical [*CrysAlis PRO* (Oxford Diffraction, 2006), based on expressions derived by Clark & Reid (1995)]	*h* = −14→14
*T*_min_ = 0.106, *T*_max_ = 0.562	*k* = −10→10
15185 measured reflections	*l* = −14→14

### Refinement

**Table:**

Refinement on *F*^2^	0 constraints
Least-squares matrix: full	Primary atom site location: structure-invariant direct methods
*R*[*F*^2^ > 2σ(*F*^2^)] = 0.017	Secondary atom site location: difference Fourier map
*wR*(*F*^2^) = 0.037	*w* = 1/[σ^2^(*F*_o_^2^) + (0.008*P*)^2^ + 3.5409*P*] where *P* = (*F*_o_^2^ + 2*F*_c_^2^)/3
*S* = 1.2	(Δ/σ)_max_ = 0.001
2388 reflections	Δρ_max_ = 0.67 e Å^−^^3^
178 parameters	Δρ_min_ = −0.69 e Å^−^^3^
0 restraints	

### Special details

**Table:**

Geometry. All e.s.d.'s (except the e.s.d. in the dihedral angle between two l.s. planes) are estimated using the full covariance matrix. The cell e.s.d.'s are taken into account individually in the estimation of e.s.d.'s in distances, angles and torsion angles; correlations between e.s.d.'s in cell parameters are only used when they are defined by crystal symmetry. An approximate (isotropic) treatment of cell e.s.d.'s is used for estimating e.s.d.'s involving l.s. planes.
Refinement. Refinement of *F*^2^ against ALL reflections. The weighted *R*-factor *wR* and goodness of fit *S* are based on *F*^2^, conventional *R*-factors *R* are based on *F*, with *F* set to zero for negative *F*^2^. The threshold expression of *F*^2^ > σ(*F*^2^) is used only for calculating *R*-factors(gt) *etc*. and is not relevant to the choice of reflections for refinement. *R*-factors based on *F*^2^ are statistically about twice as large as those based on *F*, and *R*- factors based on ALL data will be even larger.

### Fractional atomic coordinates and isotropic or equivalent isotropic displacement parameters (Å^2^)

**Table:**

	*x*	*y*	*z*	*U*_iso_*/*U*_eq_	
Lu	0.293348 (13)	0.003290 (17)	0.709429 (13)	0.00494 (5)	
Rb1	0.44695 (5)	0.75	0.54955 (5)	0.01305 (11)	
Rb2	0.08212 (5)	0.25	0.07908 (5)	0.01314 (11)	
Rb3	−0.00057 (5)	0.75	0.52863 (5)	0.01827 (13)	
Rb4	0.46419 (5)	0.25	0.03113 (5)	0.01750 (12)	
Si1	0.38671 (9)	−0.06260 (11)	0.22969 (8)	0.00464 (19)	
Si2	0.01773 (9)	0.06858 (11)	0.75550 (9)	0.00508 (19)	
Si3	0.24334 (9)	0.43354 (11)	0.38343 (8)	0.00493 (19)	
Si4	0.22493 (9)	0.93232 (11)	0.96450 (8)	0.00543 (19)	
O1	0.4021 (3)	0.75	0.2204 (3)	0.0099 (8)	
O2	0.7259 (3)	0.75	0.6326 (4)	0.0129 (8)	
O3	0.2743 (2)	0.9946 (3)	0.1053 (2)	0.0084 (5)	
O4	0.3423 (2)	0.9684 (3)	0.3435 (2)	0.0100 (5)	
O5	−0.0274 (3)	0.25	0.7485 (3)	0.0112 (8)	
O6	−0.1052 (2)	0.9599 (3)	0.7182 (2)	0.0090 (5)	
O7	0.0945 (2)	0.0289 (3)	0.9002 (2)	0.0084 (5)	
O8	0.1833 (3)	0.75	0.9630 (3)	0.0100 (8)	
O9	0.5102 (2)	0.0289 (3)	0.2481 (2)	0.0110 (5)	
O10	0.0957 (2)	0.0320 (3)	0.6743 (2)	0.0096 (5)	
O11	0.3210 (2)	0.9616 (3)	0.9020 (2)	0.0095 (5)	
O12	0.2534 (3)	0.0222 (3)	0.5157 (2)	0.0156 (6)	
F1	0.2695 (3)	0.75	0.6782 (3)	0.0142 (7)	
F2	0.3190 (3)	0.25	0.7478 (3)	0.0172 (7)	

### Atomic displacement parameters (Å^2^)

**Table:**

	*U*^11^	*U*^22^	*U*^33^	*U*^12^	*U*^13^	*U*^23^
Lu	0.00544 (8)	0.00452 (8)	0.00516 (8)	0.00016 (5)	0.00231 (6)	0.00015 (5)
Rb1	0.0146 (3)	0.0120 (2)	0.0119 (2)	0	0.0043 (2)	0
Rb2	0.0170 (3)	0.0114 (2)	0.0131 (2)	0	0.0080 (2)	0
Rb3	0.0248 (3)	0.0133 (3)	0.0122 (3)	0	0.0016 (2)	0
Rb4	0.0143 (3)	0.0133 (3)	0.0212 (3)	0	0.0024 (2)	0
Si1	0.0050 (5)	0.0044 (4)	0.0044 (4)	0.0007 (4)	0.0015 (4)	0.0002 (4)
Si2	0.0043 (5)	0.0047 (5)	0.0053 (4)	0.0002 (4)	0.0007 (4)	0.0003 (4)
Si3	0.0049 (5)	0.0052 (5)	0.0044 (4)	−0.0007 (4)	0.0013 (4)	−0.0001 (4)
Si4	0.0055 (5)	0.0057 (5)	0.0042 (4)	0.0006 (4)	0.0009 (4)	0.0007 (4)
O1	0.0112 (19)	0.0054 (17)	0.0149 (19)	0	0.0071 (16)	0
O2	0.0094 (19)	0.0051 (17)	0.021 (2)	0	0.0015 (16)	0
O3	0.0070 (12)	0.0113 (13)	0.0050 (12)	0.0022 (10)	0.0001 (10)	−0.0006 (10)
O4	0.0100 (13)	0.0113 (13)	0.0101 (12)	0.0043 (10)	0.0053 (11)	−0.0006 (10)
O5	0.0093 (19)	0.0069 (18)	0.0156 (19)	0	0.0027 (16)	0
O6	0.0048 (12)	0.0099 (12)	0.0097 (12)	−0.0021 (10)	−0.0002 (10)	−0.0001 (10)
O7	0.0074 (13)	0.0118 (13)	0.0063 (12)	0.0036 (10)	0.0029 (10)	−0.0007 (10)
O8	0.0134 (19)	0.0035 (17)	0.0127 (18)	0	0.0045 (16)	0
O9	0.0082 (13)	0.0091 (13)	0.0145 (13)	−0.0019 (10)	0.0028 (11)	−0.0004 (11)
O10	0.0084 (13)	0.0127 (13)	0.0076 (12)	0.0016 (10)	0.0030 (10)	0.0011 (10)
O11	0.0089 (13)	0.0137 (13)	0.0069 (12)	0.0010 (10)	0.0042 (10)	0.0023 (10)
O12	0.0179 (15)	0.0221 (15)	0.0075 (12)	0.0039 (11)	0.0054 (11)	0.0023 (11)
F1	0.0164 (17)	0.0044 (14)	0.0194 (17)	0	0.0039 (14)	0
F2	0.0220 (18)	0.0056 (15)	0.0250 (18)	0	0.0100 (15)	0

### Geometric parameters (Å, º)

**Table:**

Si1—O9	1.581 (3)	Rb1—O4^xi^	3.332 (3)
Si1—O1^i^	1.6157 (11)	Rb2—O7^xii^	2.875 (2)
Si1—O4^i^	1.631 (3)	Rb2—O7^xiii^	2.875 (2)
Si1—O3^i^	1.640 (2)	Rb2—O6^iii^	2.921 (3)
Si2—O10	1.579 (3)	Rb2—O6^xiv^	2.921 (3)
Si2—O6^i^	1.625 (3)	Rb2—O8^xiv^	2.949 (4)
Si2—O5	1.6259 (14)	Rb2—O3^iv^	3.058 (2)
Si2—O7	1.645 (3)	Rb2—O3^i^	3.058 (2)
Si3—O12^ii^	1.569 (3)	Rb2—O7^xv^	3.214 (3)
Si3—O6^iii^	1.630 (3)	Rb2—O7^xvi^	3.214 (3)
Si3—O4^iv^	1.631 (3)	Rb2—O2^v^	3.312 (4)
Si3—O2^v^	1.6318 (15)	Rb3—O10^xvi^	2.909 (3)
Si4—O11	1.574 (3)	Rb3—O10^xiv^	2.909 (2)
Si4—O8	1.6264 (14)	Rb3—O10^vii^	2.927 (3)
Si4—O3^vi^	1.634 (3)	Rb3—O10^ii^	2.927 (3)
Si4—O7^vii^	1.648 (3)	Rb3—F1	2.988 (3)
Lu—F2	2.1487 (7)	Rb3—O12^xvi^	3.407 (3)
Lu—O12	2.167 (3)	Rb3—O12^xiv^	3.407 (3)
Lu—O9^viii^	2.175 (3)	Rb3—O5^xiv^	3.409 (4)
Lu—F1^i^	2.1906 (6)	Rb3—O6	3.426 (3)
Lu—O10	2.200 (2)	Rb3—O6^iv^	3.426 (3)
Lu—O11^i^	2.205 (2)	Rb4—O11^xi^	2.949 (3)
Rb1—O9^v^	2.938 (3)	Rb4—O11^v^	2.949 (3)
Rb1—O9^ix^	2.938 (3)	Rb4—O11^xvii^	3.045 (3)
Rb1—O4^iv^	2.947 (3)	Rb4—O11^xviii^	3.045 (3)
Rb1—O4	2.947 (3)	Rb4—O9^ii^	3.065 (3)
Rb1—F1	2.989 (3)	Rb4—O9	3.065 (3)
Rb1—O2	3.031 (4)	Rb4—F2^xii^	3.142 (3)
Rb1—O12^ii^	3.158 (3)	Rb4—O3^iv^	3.444 (3)
Rb1—O12^vii^	3.158 (3)	Rb4—O3^i^	3.444 (3)
Rb1—O4^x^	3.332 (3)		
			
F2—Lu—O12	96.25 (12)	O6^i^—Si2—O7	104.51 (14)
F2—Lu—O9^viii^	91.29 (11)	O5—Si2—O7	106.84 (17)
O12—Lu—O9^viii^	92.79 (10)	O12^ii^—Si3—O6^iii^	112.89 (15)
F2—Lu—F1^i^	177.69 (12)	O12^ii^—Si3—O4^iv^	111.44 (15)
O12—Lu—F1^i^	86.05 (11)	O6^iii^—Si3—O4^iv^	109.22 (14)
O9^viii^—Lu—F1^i^	88.38 (11)	O12^ii^—Si3—O2^v^	114.08 (18)
F2—Lu—O10	89.16 (11)	O6^iii^—Si3—O2^v^	104.29 (16)
O12—Lu—O10	89.57 (10)	O4^iv^—Si3—O2^v^	104.34 (17)
O9^viii^—Lu—O10	177.53 (9)	O11—Si4—O8	114.10 (17)
F1^i^—Lu—O10	91.08 (11)	O11—Si4—O3^vi^	112.49 (14)
F2—Lu—O11^i^	89.10 (11)	O8—Si4—O3^vi^	108.21 (17)
O12—Lu—O11^i^	173.81 (10)	O11—Si4—O7^vii^	113.66 (14)
O9^viii^—Lu—O11^i^	90.18 (9)	O8—Si4—O7^vii^	104.55 (16)
F1^i^—Lu—O11^i^	88.61 (11)	O3^vi^—Si4—O7^vii^	102.93 (13)
O10—Lu—O11^i^	87.40 (9)	Si1^vii^—O1—Si1^ii^	162.7 (3)
O9—Si1—O1^i^	112.24 (17)	Si3^ix^—O2—Si3^v^	146.9 (3)
O9—Si1—O4^i^	110.99 (14)	Si4^xii^—O3—Si1^vii^	132.77 (16)
O1^i^—Si1—O4^i^	107.06 (17)	Si3^iv^—O4—Si1^vii^	142.83 (17)
O9—Si1—O3^i^	111.24 (14)	Si2^ii^—O5—Si2	144.0 (2)
O1^i^—Si1—O3^i^	107.75 (16)	Si2^vii^—O6—Si3^xvi^	144.19 (17)
O4^i^—Si1—O3^i^	107.32 (13)	Si2—O7—Si4^i^	129.48 (16)
O10—Si2—O6^i^	111.85 (14)	Si4—O8—Si4^iv^	145.7 (3)
O10—Si2—O5	113.95 (17)	Lu^vii^—F1—Lu^ii^	160.51 (16)
O6^i^—Si2—O5	106.95 (16)	Lu—F2—Lu^ii^	156.30 (18)
O10—Si2—O7	112.13 (14)		

Symmetry codes: (i) *x*, *y*−1, *z*; (ii) *x*, −*y*+1/2, *z*; (iii) −*x*, *y*−1/2, −*z*+1; (iv) *x*, −*y*+3/2, *z*; (v) −*x*+1, −*y*+1, −*z*+1; (vi) *x*, *y*, *z*+1; (vii) *x*, *y*+1, *z*; (viii) −*x*+1, −*y*, −*z*+1; (ix) −*x*+1, *y*+1/2, −*z*+1; (x) −*x*+1, −*y*+2, −*z*+1; (xi) −*x*+1, *y*−1/2, −*z*+1; (xii) *x*, *y*, *z*−1; (xiii) *x*, −*y*+1/2, *z*−1; (xiv) −*x*, −*y*+1, −*z*+1; (xv) −*x*, −*y*, −*z*+1; (xvi) −*x*, *y*+1/2, −*z*+1; (xvii) *x*, *y*−1, *z*−1; (xviii) *x*, −*y*+3/2, *z*−1.
